# The association between burnout, perceived organizational support, and perceived professional benefits among nurses in China

**DOI:** 10.1016/j.heliyon.2024.e39371

**Published:** 2024-10-18

**Authors:** Huifang Zhang, Victor Chee Wai Hoe, Li Ping Wong

**Affiliations:** a Department of Social and Preventive Medicine, Faculty of Medicine, University Malaya, 50603 Kuala Lumpur, Malaysia; b Center for Population Health (CePH), Department of Social and Preventive Medicine, Faculty of Medicine, Universiti Malaya, Kuala Lumpur, 50603 Malaysia; c Department of Epidemiology and Health Statistics, School of Public Health, Fujian Medical University, Fuzhou 350122 Fujian, China; d Department of Medicine, College of Medicine, Korea University, 145 Anam-ro, Seongbuk-gu, Seoul 02841 Republic of Korea

**Keywords:** burnout, Perceived organizational support, Perceived professional benefits, correlation, Regression, nurses

## Abstract

**Objective:**

This study sought to investigate the relationship between burnout, perceived organizational support (POS) and perceived professional benefits (PPB)as well as to explore factors associated with burnout among nurses in Chinese public hospitals.

**Methods:**

The purposive sampling method was used to collect data from May 2023 through August 2023. A total of 1058 nurses from 5 public hospitals in Beijing, China, completed the questionnaires. The study used a cross-sectional survey design with data obtained using the Maslach Burnout Inventory (MBI-GS), Perceived Organizational Support Scale, and Perceived Professional Benefits Scale. Pearson correlation was used to examine the association between burnout and perceived organizational support with perceived professional benefits, while multivariate regression was used to investigate the specific factors related to both perceived organizational support and perceived professional benefits influencing burnout.

**Results:**

Effective response rate was 1058(93.34 %). The total mean score of burnout was (3.024 ± 0.711), range = (1.59–4.50). Pearson correlation showed that the total mean score of burnout was negatively correlated with the total mean score of POS (r = −0.223, *p* < 0.01. Additionally, the total mean score of burnout showed a negative correlation with the total mean score of PPB. Multiple linear regression analysis showed that affective perceived organizational support (*p* < 0.01) and superior support (*p* < 0.01), sub-dimensions of perceived organizational support, were the main factors influencing burnout. There were no demographic disparities in burnout, except that nurses with ≤8 shift hours per week at night had significantly higher burnout scores (3.19 ± 0.72).

**Conclusions:**

The study's participants experienced moderately high level of burnout. the findings highlight perceived organizational support and perceived professional benefit can be critical in alleviating burnout, medical institutions and nursing managers should undertake timely and effective interventions to boost organizational support and improve the degree of benefit from their profession. It is also recommended that adequate organizational support be prioritized, with a primary focus on improving affective organizational support and strengthening supervisor support.

## 1 Introduction

Burnout among nursing professionals has become a critical issue affecting healthcare systems worldwide. Burnout is a complex manifestation of physical and mental exhaustion as well as energy depletion resulting from prolonged exposure to work-related stress [1]. Existing studies have employed various methods to define and measure burnout. One of the most widely used instruments is the Maslach Burnout Inventory (MBI), which evaluates the three dimensions of burnout: emotional exhaustion, personality disintegration, and occupational fulfillment (Leiter & Maslach, 2009; [2]). Emotional exhaustion refers to feelings of being overextended and depleted of emotional and physical resources; personality disintegration is characterized by negative, callous, or detached responses to various aspects of the job; and occupational fulfillment is the feeling of competence in work achievement and productivity. The concept of burnout was first introduced by Freudenberger in the 1970s and has since been extensively studied across various professional contexts, with a significant focus on the healthcare sector. Previous research consistently shows high levels of burnout among nurses due to factors such as long working hours, high patient-to-nurse ratios, and the emotional demands of patient care [3]. Nurses experiencing burnout may lose patience and affection for patients, have diminished control and achievement, which impacts not only their own mental and physical health but also the entire nursing team and organizational development [4,5].Burnout among nurses in China is particularly severe, with incidence rates ranging from 55.1 % to 59.1 % [6], and reports from some provinces reaching as high as 62.8 % [7]. Therefore, more research needs to be carried out to understand factors influencing burnout.

Boamah et al. [8] revealed that burnout might be related to several factors, including leadership ability in building strong perceived organizational support, organizational rewards, and work-life interference. These factors are conducive to improving positive emotions [9,10]. POS refers to employees’ overall perception that organization values their contributions and cares about their well-being [11]. Eisenberger et al. [12] introduced the concept, and it has since been measured using perceived organizational support scale. It consists affective perceived organizational support, instrumental organizational support, superior support and colleague support (Chen,2006). POS decreases the psychological and mental responses to stress by indicating the effectiveness of material and emotional support to meet higher job demands [13]. Increasing POS for caregivers can alleviate burnout and reduce turnover rates [14]. Past research has indicated that organizational support is a significant factor influencing burnout among nurses [15], and evidence has also shown that higher levels of organizational support may reduce the impact of different workplace stressors (Veenema et al., 2023). Robust POS can effectively alleviate the work pressure experienced by employees, including nurses, and mitigate job burnout. Huiyang Sheng (2023) reported perceived organizational support to be negatively associated with a general measure of burnout. Han Chen [16] found a negative correlation between perceived organizational support and emotional exhaustion in restaurant industry. In Italy, Capozza (2003) showed the negative correlation between perceived organizational support, emotional exhaustion, and cynicism components in job burnout. However, according to literature, there has been limited research attention on organizational support in the job burnout (Zhihua Xu,2018),only a few studies have investigated the relationship between POS and burnout [17,18] among nurses in China.

Above mentioned factors potentially increase nurses’ fatigue and burnout. Furthermore, studies have found that PPB is an important factor that stimulates the inner potential of nurses (Chen ZH,2011). Previous studies confirmed that PPB plays an important role in alleviating emotional exhaustion among nurses ([19] their profession in the process of the nursing practice and agree that the nursing profession could promote their overall growth [20], which contains job security, medical resources, and work value aspects. Zhang [null] fo und that enhancing the professional satisfaction of oncology nurses helps them cope with the cumulative losses and grief encountered in clinical practice. Additionally, Cheng found that PPB can reduce burnout among pediatric nurses [21], suggesting a correlation between PPB and burnout [22]. Domestic studies on perceived professional benefits have primarily focused on its status. The exploration of the relationship between PPB and burnout is still in its early stages [null]. Therefore, gaining a thorough understanding of how PPB affects burnout is essential to clarify their relationship. This understanding can also provide directions and references for improving PPB, reducing burnout, and fostering the stability and the development of nursing teams. Based on the above evidence, another question with this study is PPB and sub-dimensions are associated with burnout among nurses in China.

To date, evidence of the relationship between burnout, POS, and PPB in China is limited. Additionally, factors associated with burnout among nurses are not well understood and require further investigation. The main objective of this study is to investigate the relationship between burnout, POS and PPB. Secondly, this study also explores factors associated with burnout among nurses in Chinese public hospitals. This study takes a novel approach by examining the interplay between external POS and internal PPB to shed light on how they jointly influence burnout. Findings potentially inform targeted implementation of strategies in healthcare organizations to reduce burnout, improve POS, and ultimately, provide high-quality patient care strategies while ensuring PPB for nursing staff.

## 2 Methods and procedure

### 2.1 Design

This study utilized a cross-sectional design and adhered to the STROBE guidelines [23].

### 2.2 Sampling selection

This study was a cross-sectional survey conducted in five public hospitals in Beijing, China, from May to August 2023. The geographical scope of the study was confined to Beijing, the capital city, which has the highest concentration of hospitals and healthcare personnel. Hospitals were selected from five major administrative districts: Dongcheng District, Xicheng District, Chaoyang District, Haidian District, and Fengtai District. By selecting one hospital per district, the sample ensured coverage across various parts of the city. And the hospitals in the sample are university-affiliated, each with over 700 beds, providing medical services, education, research, prevention, health care, and recovery. The nursing staff at each hospital comprises more than 600 employees. These hospitals are similar in terms of the number of nurses, hospital size, type, working conditions, management, economic income.

Regarding sample size, G Power 3.1 software was used to conduct a power analysis for MANOVA and to calculate the sample size [24]. The sample size estimation with G Power 3.1 software was 713 cases. Accounting for a 10 % margin for inaccurate questionnaires, a minimum sample size of approximately 800 was required. This study successfully achieved the necessary sample size.

Lastly, based on the desired overall sample size, a proportionate sample size was allocated to each hospital based on its contribution to the total number of nurses. The weight for each hospital and the expected sample size for each hospital were calculated. N_i_ is the number of nurses at hospital i, N is the total number of nurses across all hospitals, and N_total_ is the total desired sample size for the study. Subsequently, the sample sizes for each hospital were 192,168,145,152, and 143, respectively, resulting in a total sample of 800.

### 2.3 Participants

The inclusion criteria were nurses with professional certification and possessing a minimum of one-year continuous nursing experience; exclusion criteria were nurses on a training rotation or internship. The study implemented stringent quality control measures. The estimated average time for completing the survey was 10–15 min. The study monitored response times, and surveys completed in less time than the specified limit were removed. To avoid data duplication and fraud, measures such as restricting repeated IP address was employed. Additionally, cash incentive was given to help reduce nonresponse bias. Incentives also help to improve the representativeness of the sample and reduce the likelihood of certain groups being underrepresented. Furthermore, the use of anonymous survey reduces social desirability bias, and this helps in obtaining more accurate and honest responses. Reverse-coding questions was used to reduce response bias and improve accuracy of responses by ensuring that participants are paying attention and carefully considering their answers. Consequently, straight-lining responses were removed to further enhance data reliability. To ensure data completeness, a prompt is activated when submitting the questionnaire. If any responses are incomplete, the survey cannot be submitted, and the prompt will alert participants about the unanswered questions. Participants will be directed to complete these questions before they can successfully submit the survey. After eliminating invalid responses, 1058 questionnaires were deemed suitable for statistical analysis, resulting in a response rate of 93.34 %. Given that the calculated sample size was 800, the inclusion of 1058 nurses met this requirement.

### 2.4 Survey instruments

The survey instruments included a demographic questionnaire, the Maslach Burnout Inventory – General Survey (MBI-GS), the POS scale, and the PPB scale.

### 2.5 General demographic questionnaire

The questionnaire to obtain demographic data was created based on previous research on burnout [18]. Demographic variables included gender, age, marital status, highest academic qualification, and work characteristics such as job title, work unit, type of employment, years of service, type of night shift, weekly night shift hours, weekly number of night shifts, and average number of patients seen by the participants per day.

### 2.6 Burnout scale

This study assess burnout useing the Chinese version of the MBI instrument. Chapping Lee et al. (2003) translated the instrument based on Maslach and Jackson [25]. The instrument has 22 items that assess three dimensions: emotional exhaustion (EE; 9 items), personality disintegration (PD; 5 items), and occupational fulfillment (OF; 8 items). Items in the EE dimension evaluate the emotional response to occupational stress. While thosein the PD dimension assess the stress-induced attitudes and feelings toward the service recipients. Items for EE and PD have positive scores—the higher the score, the more severe the burnout. The items for OF evaluate the stress-induced perceptions of one's own work; this is scored in reverse, which means that the lower the score, the worse the burnout. To help participants understand the notion, the individual items for the OF dimension were positively treated, meaning that the higher the score, the more severe the burnout. The scale is rated on a 5-point Likert scale, ranging from 1 (*low level of agreement*) to 5 (*high level of agreement*). Again, higher scores indicate a higher level of burnout. The Cronbach's coefficient for the scale in this study was 0.923. Validation factor analysis shows good fit results (χ^2^/df = 2.087; NFI = 0.968; CFI = 0.983; RMSEA = 0.032; SRMR = 0.048).

### 2.7 POS scale

Chen [26] amended the POS Scale based on Eisenberger [12], and the proposed four-dimensional structure of POS was taken as reference for translating a POS scale for nurses. The POS scale included 16 items based on the concepts of relatively narrow organizational support, as well as superior support and colleague support, specifically expressed in a four-dimensional structure: affective perceived organizational support (APOS, 7 items), instrumental organizational support (3 items), superior support (3 items), and colleague support (3 items). A 5-point Likert scale was employed with responses ranging from 1 (*very disagree*) to 5 (*strongly agree*). Total scores ranged between 16 and 80 points, with higher scores indicating higher levels of POS. The Cronbach's coefficient for the scale in this study was 0.876. Validation factor analysis shows good fit results (χ^2^/df = 2.095; NFI = 0.975; CFI = 0.987; RMSEA = 0.032; SRMR = 0.038).

### 2.8 PPB scale

by Xiao et al. [27] developed the Chinese version of the nurse's PPB scale based on f Hu and Liu ‘s(2013) research. The instrument has 15 items assess three dimensions: job security (JS, 3 items), medical resources (MR, 5 items), and work value (WV, 7 items). A 5-point Likert scale was used, and the responses ranged from 1 (*totally disagree*) to 5 (*absoulutely agree*). Total scores were between 15 and 75 points, with higher scores implying a higher degree of PPB. We conducted comprehensive analyses to assess the reliability and validity of the scale. The Cronbach's coefficient for the scale in this study was 0.910. Validation factor analysis showed good fit results (χ^2^/df = 2.928; NFI = 0.975; CFI = 0.983; RMSEA = 0.043; SRMR = 0.049).

### 2.9 Data Collection methods

The survey was disseminated through QuestionStar, a widely used academic online survey platform [28]. The designated contact person at each hospital distributed the QR code to eligible nurses in the department using the established standard procedures developed for the study. Participation was completely voluntary.

### Ethical approval

Ethical approval to conduct this study was granted by Research Ethics Committee of the authors’ affiliate university (UM.TNC2/UMREC_2374).the purpose and significance of the study was explained in writing at the beginning of the questionnaire, all participants were told that their responses were completely anonymous, they voluntarily completed the questionnaire. The consent form also included details the expected duration of participation, a statement emphasizing that participation was voluntary, confidentiality, and the right to withdraw from the survey at any time without needing to provide an explanation. Additionally, contact information of the research team was provided in case participants had any questions or concerns. The procedures were conducted per the ethical standards of the 1964 Declaration of Helsinki.

### 2.10 Statistical analysis

The data were analyzed with SPSS 21.0 software. Kolmogorov-Smirnov was used to evaluate data for normal distribution (p > 0.05); Cronbach's coefficient was used to test the scale's internal consistency reliability, and the scale's structural validity analysis was tested using factor analysis.

Measurement data were presented as means and standard deviations, while enumerated data were presented as frequencies and percentages. The univariate analysis between sample characteristics and variables was evaluated by an independent sample *t*-test and chi-squared test. Pearson correlation was utilized to evaluate the association between burnout, POS, and PPB, while multivariate regression was employed to analyze the influencing factors from POS with PPB on burnout. The test level α = 0.05, *p* < 0.05 was considered statistically significant. Using R version 4.2.3 to draw a diagonal correlation matrix plot, each cell in the plot shows the correlation coefficient between two variables, with the color indicating the strength of the correlation (for example, warm colors indicate positive correlation, and cool colors indicate negative correlation).

In selecting control variables, demographic factors significantly associated with burnout were included in the regression analysis.

## 3 Results

### 3.1 Demographic information

A total of 1058 study participants completed the questionnaire, for a response rate of 93.34 %. the majority of participants were females (94.3 %). More than half of them were aged between the ages of 25 and 35 years (66.9 %); with more than half were unmarried (62.8 %). The majority had at least an undergraduate qualification (70.8 %), with ≤10 years of service (60.1 %), and at least a middle level job (66.9 %). Just over half worked as contract workers (59.4 %), while just under half of the participants were internists (42.06 %). With regards to work schedule and responsibilities, 61.1 % worked a single night duty, 65.5 % were engaged in night shifts lasting 9–24 h, 56.1 % worked a three-night shift schedule, and 89.70 % were in charge of an average daily patient load of less than 10 patients (Table 1).

**Table 1:** General demographic characteristics (N = 1058).

Variables	N	%
Gender		
Female	998	94.33
Male	60	5.67
Age		
≤25	122	11.53
26-30	296	27.98
31-35	290	27.41
36-40	196	18.53
41-45	120	11.34
≥46	34	3.21
Marital status		
Married	346	32.70
Unmarried	664	62.76
Divorced/Separated/Widowed	48	4.54
Highest academic qualification		
Others	14	1.32
junior college	296	27.98
Degree	666	62.95
Post Graduate (Master/PhD)	82	7.75
Years of Service		
≤5	242	22.87
6-10	394	37.24
11-20	265	25.05
≥21	157	14.84
Type of employment		
labor contract	393	37.15
contract	628	59.36
Third party dispatch	37	3.50
Job Title		
junior	350	33.08
middle	506	47.83
senior	202	19.09
Work Unit		
Medicine	445	42.06
Surgery	373	35.26
Emergency	79	7.47
pediatric	51	4.82
Tech	34	3.21
obstetrics	34	3.21
administration	30	2.84
others	12	1.13
Night shift		
Three shifts.	594	56.14
Two shifts.	348	32.89
Others	116	10.96
Weekly night shift hours		
≤8	228	21.55
9-24	693	65.50
≥25	137	12.95
Weekly No. night shift		
1	646	61.06
2	348	32.89
≥3	64	6.05
Average No. patients per day		
≤10	949	89.70
＞11	109	10.30

Descriptive analysis on burnout, POS, and PPB.

The participants had a mean burnout score of 3.024 ± 0.711 (range = 1.59–4.5), a mean POS score of 2.809 ± 0.677 (range = 1.5–4.5), and a mean PPB score of 2.963 ± 0.787 (range = 1.07–4.8; Table 2).

**Table 2:** Characteristic findings for burnout and dimensions(N = 1058).

Variables	Dimensions	Items	Max	Min	SD(x ± s)
Burnout	Total	22	4.5	1.59	3.024 ± 0.711
	EE	9	4.89	1.11	3.015 ± 0.899
	LOF	8	4.88	1.13	3.006 ± 0.902
	PD	5	5	1	3.072 ± 0.947
POS	Total	16	4.5	1.5	2.809 ± 0.677
	APOS	7	4.86	1	2.801 ± 0.903
	IPOS	3	5	1	2.789 ± 1.019
	SS	3	5	1	2.831 ± 0.999
	CS	3	5	1	2.831 ± 1.010
PPB	Toal	15	4.8	1.07	2.963 ± 0.787
	JS	3	5	1	2.924 ± 1.097
	MR	5	5	1	2.990 ± 1.005
	WV	7	4.86	1	2.961 ± 0.954

∗ POS: perceived organizational support, PPB: perceived professional benefits.

### 3.2 Univariate analysis of burnout

There were statistically significant differences in terms of weekly night shift hours. Nurses working ≤8 shift hours per week at night had higher burnout scores (3.19 ± 0.72) compared to other groups (*p* < 0.05). There were no statistically significant differences in total scores for demographic and sociological characteristics such as gender, age, department, and marital status (Table 3).

**Table 3:** Univariate analysis of burnout(N = 1058).

	**Variable**	**N**	**Mean**	**F**	**P**
weekly night shift hours	≤8	228	3.19 ± 0.72		
	9–24	693	2.98 ± 0.71	8.199	<0.001^a^
	≥25	137	2.97 ± 0.67		

∗p < 0.05.

a p < 0.01.

### 3.3 Correlation analysis between burnout, POS, and PPB

**Fig. 1:**
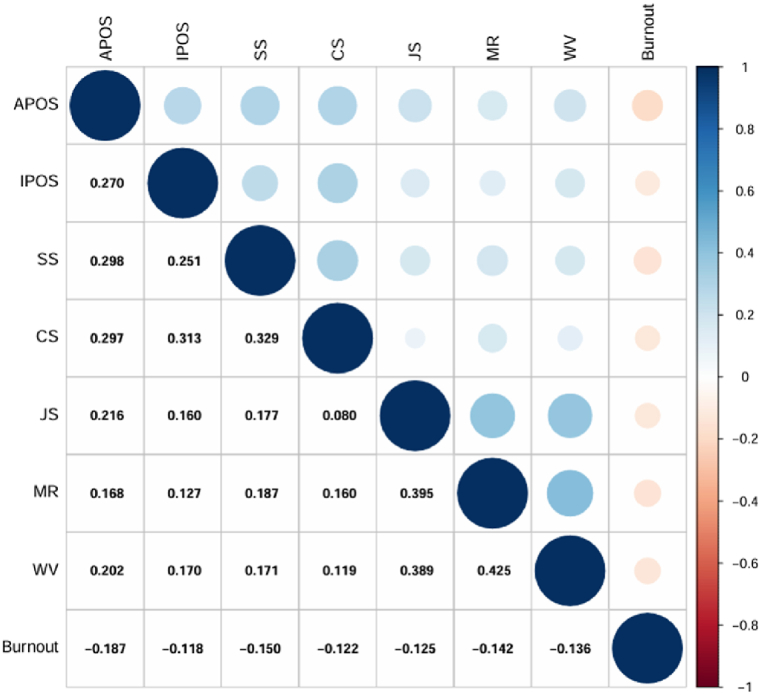
Correlation Matrix heatmap between burnout, perceived organizational support and perceived professional benefits (N = 1058). APOS: affective perceived organizational support, IPOS: instrumental organizational spport, SS: superior support, CS: colleague support, JS: job security, MR: medical resources, WV: work value.

There was a significant negative correlation between burnout and POS (r = −0.223, *p* < 0.001), PPB (r = −0.172, *p* < 0.001, Table 4), as well as all dimensions of POS and PPB (*p* < 0.001, Fig. 1).

**Table 4:** Correlation analysis between burnout, perceived organizational support and perceived professional benefits (N = 1058).

**Variable**	**1**	**2**	**3**
Burnout	1		
POS	−0.223^a^	1	
PPB	−0.172^a^	0.310^a^	1

∗p < 0.05. POS:perceived organizational support, PPB: Perceived professional benefits.

a p < 0.01.

### 3.4 Regression analysis of burnout, POS, and PPB

The results indicated that only affective perceived organizational support and superior support differed significantly (*p* < 0.05). The correlation between affective perceived organizational support dimension of POS and burnout was the strongest (−0.117), secondly, superior support dimension of POS (−0.068, Table 5). The weekly night shift hours showed no statistically significant difference.

**Table 5:** Regression analysis of burnout, perceived organizational support and perceived professional benefits (N = 1058).

**Variable**	**β**	**SE**	**St.β**	**T**	**P**
Constant	3.818	0.118	–	32.358	<0.001^a^
Weekly night shift hours	0.033	0.03	0.033	1.086	0.278
APOS	−0.092	0.026	−0.117	−3.533	<0.001^a^
IPOS	−0.026	0.023	−0.038	−1.162	0.245
SS	−0.048	0.024	−0.068	−2.056	0.040∗
CS	−0.025	0.024	−0.036	−1.068	0.286
JS	−0.022	0.022	−0.034	−0.997	0.319
MR	−0.044	0.025	−0.063	−1.811	0.070
WV	−0.035	0.026	−0.047	−1.37	0.171

∗p < 0.05, APOS: affective organizational support, IPOS: instrumental organizational spport, SS: superior support, CS: colleague support, JS: job security, MR: medical resources, WV: work value.

a p < 0.01.

## 4 Discussion

### 4.1 Current status of burnout among Chinese nurses

The findings verified the correlation between burnout and POS with PPB. A study of 1058 nurses found a moderately high level of burnout (mean score 3.024 ± 0.711). This discovery emphasizes the prevalence of burnout in the contemporary nursing profession and is consistent with the results of other scholars [29]. One probable explanation is that the study was conducted after China's quarantine policy was lifted on January 8, 2023. On April 12, the National Disease Control Administration officially rescinded the required mask regulation nationwide. Positive tests findings for COVID-19 gradually increased beginning in late April, culminating in a second peak following China's total reopening. The number of patients visiting hospitals thus significantly increased (CDC, 2023), The number of patients visiting hospitals thus increased significantly (CDC, 2023), resulting in a significant increase in the exertion and stress experienced by nurses, as well as increased concern about the uncertainties inherent in nursing duties in the face of infectious hazards. This could have made nurses more susceptible to burnout (Hai-Ping Zhang, 2023). Furthermore, due to regional and population differences, the score of burnout may differ slightly. This could have made nurses more susceptible to burnout (Hai-Ping Zhang, 2023). Furthermore, due to regional and population variances, the score of burnout may differ slightly [5].

### 4.2 The relationship between POS, PPB and burnout

This study discovered a strong and inverse correlation between burnout and POS. POS emerged as a pivotal external work asset that caters to the nurses' requisites for care, a sense of belonging, and intrinsic value. To a certain degree, it also mitigates the adverse ramifications of work stress and improves the ability to deal with challenges effectively. According to the theory of organizational support, when organizations provide nurses with resources in various aspects of support, nurses are more likely to respond positively even when unpleasant events occuror they experience burnout. This aligns with the findings of Irfan et al. [30], who found that nurses feel more accomplished in the presence of organizational support, while burnout appears as diminished depersonalization and emotional exhaustion. This echoes the research of scholar like Kim et al. [31], who underscore the substantial impact of nurses’ POS on burnout. POS thus emerges as a paramount determinant influencing burnout among nurses. Nurses experiencing burnout are predisposed to seek solace and support from organizational entities. This concurs with the findings of Zhu [32], who highlighted the pivotal role of POS in ameliorating burnout when nurses encounter distress. A judicious allocation of resources encompassing material provisions, emotional succor, and value affirmation can effectively empower individuals to confront and navigate environmental challenges, thereby lowering the risk of burnout. Nursing administrators should prioritize organizational support for nurses. By offering a holistic array of support, spanning material assistance, emotional nurturance, and value acknowledgment, burnout among nurses can be markedly attenuated, while enhancing their professional dedication.

This investigation also found that strong PPB in nurses correlates with an enhanced sense of accomplishment and lower incidence of personality disintegration and emotional exhaustion. This concurs with the conclusions drawn by scholats such as Di Gao [33] who posited that PPB is a paramount determinant influencing nursing burnout. PPB refers to the benefits and gains nurses perceive from their professions, according to conservation of resources theory, in an environment characterized by burnout, experiencing burnout can diminish the level of occupational benefits derive, making it challenging to sustain positive perceived. Faced with emotional turmoil, nurses engage in internal psychological construction, and they factor in the gratifications intrinsic to their vocation and the prospect of future development. This parallelism resonates with the findings of Hu and Liu [34], who discovered that recognizing professional accomplishments and opportunities for advancement may help to minimize burnout. The societal recognition and value provided on the nursing profession are inextricably linked to increased personal accomplishment. When nurses perceive that they are recognized for their endeavors by patients and ascertain that the societal needs of the nursing profession validate the significance of their existence, they show a heightened willingness to channel a greater portion of their capabilities into nursing. This manifests in positive professional mindset, which are indicative of a wholehearted dedication to their vocation.

### 4.3 Affective perceived organizational support and superior support were important factors influencing burnout

Furthermore, this study found that affective perceived organizational support, as a sub-dimension of POS, is an important factor influencing burnout. affective perceived organizational support is a barometer of the supportive milieu within hospitals [35]. The emotional facet signifies the organization's esteem for nurses' contributions, a concern for their well-being, and provision of avenues for professional advancement, while also encompassing facets like intimacy and respect. Such support plays a pivotal role in fulfilling nurses' social and psychological needs. Due to the expansive nature of large hospital organizations, effective hierarchical communication is critical. In the absence of consistent positive feedback and communication regarding nurses' job performance and expectations, inadequate interaction may ensue, reducing nurses' sense of value and engagement. This lack of recognition puts nurses under more stress, lowing their work motivation and increasing the risk of burnout. Mishra et al. [36] discovered that a lack of continuous positive feedback and communication can lead to employees felling undervalued, resulting in a lower commitment to their work. It is imperative for hospitals to establish effective communication channels to make nurses feel valued by the organization. This approach can alleviate external pressures, enhance their ability to navigate professional challenges and reduce the risk of burnout.

Moreover, Superior support, the sub-dimension of POS represents another pivotal factor affecting burnout. It indicates the support extended by supervisors to address nurses’ job-related concerns. The role of supervisors in cultivating a positive work environment is paramount, as their management approach significantly shapes the caliber of healthcare and nursing services within the entire unit [37]. The rationale for this trend could attributed to the conventional organizational culture and common leadership training in China, which frequently favors a hierarchical guidance approach. Task allocation and performance evaluations are predominantly structured as directives. Fortifying the adoption of a supportive management style by supervisors is thus imperative, as outlined by Shining Cai [38]. In Italy, the adoption of a supportive leadership style has been proven to enhance work performance, while research conducted in Sweden and Finland has substantiated the positive impact of a supportive leadership approach. These measures are likely to reduce their job burnout.

Lastly, weekly night shift hours represent another important demographic factor influencing burnout, Nurses whowork ≤8 h per week on night shifts experiencing higher level than those working more than 8 h. Possible reasons include frequently changing work hours, which can disrupt the balance work and rest time, potentially leading to increased fatigue among nurses during their shifts. Additionally, even with fewer weekly hours, dense workloads and high pressure may require nurses to handle substantial urgent tasks within a short timeframe, contributing to increased burnout. The hospital should offer flexible schedule alternatives to meet nurses' specific needs, aiding in better management of work-life balance and reduction of fatigue [39].

### 4.4 Implications for practice

Based on the findings of this study, several implications for future nursing practice can be suggested. First, the negative relationship between POS and burnout highlights the importance of organizational support, particularly in terms of affective support and superior support. Hospital administrators should establish affective support networks to provide nurses with accessible communication and feedback opportunities, while also monitoring their workload and emotional well-being. Recognizing and rewarding exceptional performance can acknowledge their hard work [40] and ensure that their emotional needs are valued. Second, nursing managers should foster supportive relationships between employees and their superiors. Superiors should enhance their support by demonstrating trust and supportive behaviors, promoting a transparent and supportive leadership philosophy within the organizational culture. Equipping supervisors with effective management skills through targeted leadership training and establishing mentorship programs can help nurses develop leadership skills and promote sustainable development [41]. Additionally, since PPB significantly impact burnout, strategies to optimize nursing benefits should be implemented. Hospitals should focus on improving nurses' professional mindset and confidence in their work to enhance their overall job satisfaction and reduce burnout.

### 4.5 Limitations

There are some theoretical limitations in this study that warrant consideration. The study might oversimplify the complexity of burnout by not fully accounting for factors beyond POS and PPB, such as personal characteristics, work-life balance, and external stressors. Therefore, current theoretical framework may not capture the nuances of these relationships, and alternative models could provide different insights. Furthermore, potential confounding variables like individual coping strategies, team dynamics, and institutional policies might not be considered, which could further limit the study's scope.These factors should be considered in future studies. Regarding methodological limitations, the study first employed self-report measures to assess burnout, POS, and PPB, which may be subject to response bias despite emphasizing anonymity and confidentiality. Additionally, the cross-sectional design restricts the ability to establish causal relationships from the findings; thus, future research should incorporate longitudinal studies to enhance the robustness of causal claims. Expanding the survey to include hospitals in other regions of China and increasing the sample size would also improve the representativeness, universality, and generalizability of the study. Therefore, findings should be interpreted with caution due to these theoretical and methodological limitations.

## 5 Conclusions

According to the findings of the present study, burnout is in the upper-middle range among nurses. Burnout is inversely related to POS and PPB, and affective perceived organizational support and superior support emerged as significant factors that influence burnout among nurses. It is suggested that some medical institution and nursing managers should implement effective interventions to boost organizational support and the degree of benefit from their profession, paying more attentionto increase support from the emotional side of hospital. For example, organizations should foster open communication channels by affective support networks, deliver timely reward regarding the significance of contributions, and enhance team morale. There is also a need to fortify and enhance supervisors’ supportive management capabilities through targeted leadership training.

## CRediT authorship contribution statement

**Huifang Zhang:** Writing – original draft. **Victor Chee Wai Hoe:** Writing – review & editing. **Li Ping Wong:** Writing – review & editing.

## Data availability statement

The datasets used and analyzed during the current study are available (https://doi.org/10.6084/m9.figshare.26801413.v1, 10.6084/m9.figshare.26801413, CC BY 4.0)

## Declaration of competing interest

The authors declare that they have no known competing financial interests or personal relationships that could have appeared to influence the work reported in this paper.

## Abbreviations

**The following abbreviations are used in this manuscript**
MBI-GS: Maslach Burnout Inventory – General Survey
POS: Perceived Organizational Support
PPB: Perceived Professional Benefits
EE: Emotional Exhaustion
PD: Personality Disintegration
OF: Occupational Fulfillment
LOF: Low Occupational Fulfillment
APOS: Affective Perceived Organizational Support
IPOS: Instrumental Perceived Organizational Support
SS: Superior Support
CS: Colleague Support
JS: Job Security
MR: Medical Resources
WV: Work Value
